# Drug resistance dependent on allostery: A P-loop rigor Eg5 mutant exhibits resistance to allosteric inhibition by STLC

**DOI:** 10.3389/fonc.2022.965455

**Published:** 2022-10-12

**Authors:** Rose-Laure Indorato, Salvatore DeBonis, Isabel Garcia-Saez, Dimitrios A. Skoufias

**Affiliations:** Université Grenoble Alpes, CNRS, CEA, Institut de Biologie Structurale (IBS), Grenoble, France

**Keywords:** mitosis, Eg5, rigor mutant, drug resistance, allostery

## Abstract

The mitotic kinesin Eg5 has emerged as a potential anti-mitotic target for the purposes of cancer chemotherapy. Whether clinical resistance to these inhibitors can arise is unclear. We exploited HCT116 cancer cell line to select resistant clones to S-trityl-L-cysteine (STLC), an extensively studied Eg5 loop-L5 binding inhibitor. The STLC resistant clones differed in their resistance to other loop-L5 binding inhibitors but remained sensitive to the ATP class of competitive Eg5 specific inhibitors. Eg5 is still necessary for bipolar spindle formation in the resistant clones since the cells were sensitive to RNAi mediated depletion of Eg5. One clone expressing Eg5(T107N), a dominant point mutation in the P-loop of the ATP binding domain of the motor, appeared to be not only resistant but also dependent on the presence of STLC. Eg5(T107N) expression was associated also with resistance to the clinical relevant loop-L5 Eg5 inhibitors, Arry-520 and ispinesib. Ectopic expression of the Eg5(T107N) mutant in the absence of STLC was associated with strong non-exchangeable binding to microtubules causing them to bundle. Biochemical assays showed that in contrast to the wild type Eg5-STLC complex, the ATP binding site of the Eg5(T107N) is accessible for nucleotide exchange only when the inhibitor is present. We predict that resistance can be overcome by inhibitors that bind to other than the Eg5 loop-L5 binding site having different chemical scaffolds, and that allostery-dependent resistance to Eg5 inhibitors may also occur in cells and may have positive implications in chemotherapy since once diagnosed may be beneficial following cessation of the chemotherapeutic regimen.

## Introduction

Even though cancer therapy has improved significantly over the last decades, it often ultimately fails due to the development of drug resistance. The phenotypic plasticity associated with the inherent genomic instability of cancer cells serves as the primary cause of intrinsic or acquired therapy resistance and therapy failure allowing tumor relapse (1). Therefore, the discovery of new drugs and new targets in the already targeted pathways are urgently needed. To this goal, the kinesin motor protein Eg5 has been actively pursued in the last three decades for the development of inhibitors of mitosis (2) as putative alternative to anti-mitotic cancer chemotherapy based on agents targeting microtubules (3).

Eg5 is a homotetrameric kinesin motor protein arranged in two antiparallel dimers able to crosslink and slide antiparallel spindle microtubules (4), an activity which is necessary for the separation of the duplicated centrosomes in early mitosis and the formation of a bipolar spindle (5). Inhibition of Eg5 activity (6) or absence of Eg5 motor following RNAi depletion in cells (7) leads to mitotic failure with a characteristic monopolar spindle phenotype due to the inability of cells to slide antiparallel microtubules nucleated by the two unseparated centrosomes (8), forcing a cell cycle arrest in mitosis due to the activation of the mitotic spindle assembly checkpoint (9). The faith of the arrested cells appears to be cell type dependent (10, 11), but one thing is for sure, when Eg5 activity is absent or inhibited cells stop dividing normally.

Most, of the Eg5 inhibitors that entered clinical trials are allosteric in their mode of action (2). They bind to the helix α2/loop L5, and helix α3 pocket (12), which is approximately 10 Å away from the ATP binding site. Structural studies revealed that binding of a number of ligands to this pocket induces a rearrangement of loop L5, which allosterically transmits conformational changes in the ATP binding pocket, trapping the motor domain in an ATP like conformation, with ADP bound to it (13–20). As a consequence of the inhibitor binding to the α2/loop L5, and helix α3 pocket, the motor domain exhibits a low nucleotide exchange, and it can not reinitiate a new chemomechanical step. As a result, the motor activity remains inhibited as long as the inhibitor remains bound to the motor.

In the past, multiple efforts have been pursued to understand how acquired drug resistant may arise by the use of Eg5 inhibitors in cancer cells. First, the knowledge of the ligand-Eg5 motor binding pocket and extensive mutagenesis analysis in the helix α2/loop L5/helix α3 allosteric binding site of Eg5 has shown that certain amino acid substitutions can confer resistance to a variety of loop L5 inhibitors, including monastrol, S-trityl-L-cysteine (STLC), ispinesib and others (21–25). The identified amino acid substitutions in the inhibitor-binding pocket (including D130A; D130V; D133A and L214A, among others), although they do not alter appreciably the binding of monastrol or STLC to the motor domain (22), they can overcome inhibition by blocking the allosteric communication network to the ATP binding site. Furthermore, clonal selection of cells in the presence of Eg5 inhibitors such as ispinesib (26, 27), a loop L5 binding inhibitor, and BRD9647 that binds to the α4 and α6 helices (28), resulted in selection of resistant cells that express Eg5 mutants having amino acid substitutions in their respective inhibitor binding pockets. For example, the mutation D130V which is in the helix α2/loop L5/helix α3 allosteric binding site is associated with ispinesib resistance and the mutation Y104C, located in a distinct Eg5 allosteric binding site between the α4 and α6 helices associated with BRD9647 resistance.

In addition, clonal selection of cells in the presence of Eg5 inhibitors revealed another mode of resistance that was attributed to the functional plasticity of microtubule based motors substituting for loss of Eg5 function. For example, dynein and KIF15 have been shown to substitute for the loss of Eg5 function in the presence of STLC (29, 30).

In this report we used HCT116 cancer cell line to select clones that are capable of proliferating in the presence of STLC, a loop-L5 binding inhibitor of Eg5 (31). The STLC resistant clones remained sensitive to the ATP class of competitive Eg5 specific inhibitors (32) and were differentially resistant to other loop L5 binding inhibitors depending on the chemical scaffold of each inhibitor. Certain clones were not only resistant but also dependent on the presence of STLC as well as to the clinically relevant inhibitors, Arry-520 (33) and ispinesib (34–37). Dependency to STLC was associated with the expression of Eg5(T107N) mutant which when expressed in cells in the absence of the inhibitor caused strong microtubule bundling, due to a non-exchangeable association of the motor with the microtubules. Most importantly, and in contrast to the low exchange of the hydrolyzed nucleotide observed in the wild type Eg5-STLC complex, the mutant in the presence of the inhibitor retained the ability to exchange the ADP by ATP *in vitro.* We predict that resistance by dependence to allostery to Eg5 inhibitors may also occur in cells. We discuss the data in terms of their possible clinical significance.

## Material and methods

### Resistant colony isolation and colony formation assays

HCT116 p53+/+ cells were obtained from Dr. Vogelstein (Johns Hopkins University, Baltimore, MD) and were grown in McCoy medium supplemented with 10% FBS (Hyclone) and 1% L-glutamine and streptomycine/penicilline. For the selection purposes cells were plated in 15 cm plate and exposed to 10 μM STLC and the STLC (Novabiochem, Merck KGaA) containing medium was changed twice per week for 4 weeks. Following ring selection, positive clones were kept under continuous presence of STLC. Colony formation assays, flow cytometry and immunofluorescence, were performed essentially as described in (38). Dimethylenastron (EgIII), EgVI and GSK-1 (EgVII) were purchased from Santa Cruz Biotechnology Inc whereas K858, Arry-520 and ispinesib were purchased from TOCRIS Bioscience. Proliferation of HCT116 cells and cell from the selected clones was assessed using an MTT colorimetric assay (Cell Proliferation Kit I, Roche) according to the manufacture’s instructions. Cells were seeded at a concentration of 15,000 cells per well in 100 **μ**l culture medium containing each inhibitor at the indicated concentration into 96 wells microplates (Falcon ref. 353072). Plates were incubated at 37°C and 5% CO2 for 72h. Plates were allowed to stand overnight in the incubator before measuring the spectrophotometrical absorbance at 570 nm and at the reference wavelength of 690 nm in a ClarioStar plate reader. The values of A570 nm–A690 nm were normalized relative to that obtained with vehicle (0.2% DMSO). Assays in the presence of STLC were carried our in 6 replicates and the assays in the presence of rest of the inhibitors were carried out in quadruplicates; data were subjected to a null hypothesis by student’s t-test to compare each clone to the wild type.

### RNA interference

Endogenous KIF11 expression is silenced by transient transfection with two Eg5 siRNAs (Dharmacon) targeting two 3’UTR sequences NNUGAGCCUUGUGUAUAGAUU and NNUGAGCUUAACAUAGGUAAA. Transfections were carried out in 6 wells plates with 100 nM of siRNA mixed with Oligofectamine, according the manufacturer’s instructions. Following transfections of the cells, the transfection medium was replaced by fresh medium and the resistant cells were allowed to grow for two days in the presence of STLC. Naive cells in the absence of STLC were used as a control. Control and resistant cells were fixed for indirect immunofluorescence. Monopolar vs bipolar spindles were counted as described before and an unpaired samples t-test was performed to compare each clone to the untreated wild type.

### Immunofluorescence microscopy

24h after the transfection, the coverslips were washed with PBS 1X, and then fixed with a solution of 2% paraformaldehyde in PBS 1X and incubated for 20 min at 37°C. After three washing steps with PBS 1X, they were permeabilized for 3 min with 0.2% triton and then washed 3 times with PBS 1X. Coverslips were placed in a humid chamber, incubated with a solution of primary antibodies: mouse monoclonal anti-tubulin clone TUB2.1 (Sigma: T4026) diluted at 1/450, human autoantibody against centromeres HCT-0100 (*Immunovision*) diluted at 1/450, mouse monoclonal anti-myc antibody (Covance, Berkeley, CA), rabbit polyclonal anti-TPX2 (Bethyl Laboratories, Motgomery, TX) diluted at 1/200 in antibody buffer (PBS 1X, sodium azide 0,02%, Tween 0,05%, and 3% (w/v) BSA) for 1 hour at 37°C. The coverslips were then washed 3 times for 5 min by immersion in PBS 1X. Coverslips were then incubated for 30 min at 37°C in a solution containing the corresponding Alexa fluo-488 and -568 conjugated goat anti-mouse, goat anti-rabbit and goat anti-human secondary antibodies (Invitrogen), diluted 1/400 in antibody buffer. Coverslips after washing 3 times in PBS 1X for 5 min, they were deposited onto a 5 μL of VECTASHIELD mounting media deposited on slides and sealed with nail polish. VECTASHIELD was used to protect samples from photobleaching and contains DAPI, a fluorescent molecule to stain DNA.

The cells are observed using Olympus microscope (IX 80), with an objective 60X equipped with a high-sensitivity camera. Excitation and emission filter wheels and signal processing were controlled by the Volocity software.

### Purification and amplification of RNA from the resistant cells

Total RNA was isolated by using Trizol (Invitrogen) and the Nucleospin Kit^®^ RNA II (Macherey-Nagel) according to the manufacturer’s protocol, and KIF11 cDNA from the different clones was amplified by using Superscript™ One-Step RT-PCR RT/Platinum^®^ *Taq* (Invitrogen), using the following primers Eg5_372 forward 5’-GTGGTGAGATGCAGACCATTTA-3’ and Eg5_1228 reverse 5’-GGTGTTCTTTCTACAAGGGCAG-3’ and then sequenced. Site-directed mutagenesis was performed by the use of Phusion^®^ High-Fidelity DNA Polymerase (New England Biolabs) with pcDNA Myc-Eg5 as template (Blangy et al) and the following primers: T107N forward 5’-CACTATCTTTGCGTATGGCCAAAATGGCACTGGAAA-3’; T107N reverse 5’-TTTCCAGTGCCATTTTGGCCATACGCAAAGATAGTG-3’. All resulting plasmids were sequence verified.

### Construction of pGFP-C1-Eg5 WT and mutants

The resulting 3729 bp fragment, following EcoRI digestion of the RcCMV-Eg5 vector (generous gift of Anne Blangy, CRBM Montpellier France), was ligated to the EcoRI linearized pGFP-C1 vector. The correct orientation of the insert was verified by HindIII digestion and the presence of a 2800bp fragment, in the case of the correct orientation, as opposed to the 1000bp fragment in the opposite orientation. The selected plasmids were further verified for the correct orientation by sequencing. The pGFPC1-Eg5(WT) vector was used for *in vitro* mutagenesis to introduce the related mutations using the primers used above. All resulting plasmids were sequence verified.

### Fluorescence recovery after photobleaching

48h after transfection, cells grown on glass coverslips were inverted with the cell face down in 2-well Labtek chambers and the medium was changed with DMEM/F-12 supplemented with 10% fetal bovine. Cells were then imaged in the M4D Cellular Imaging platform of IBS with a spinning disk confocal microscope (Olympus and Andor) configurated with a Nipkow wheel (Yogokawa CSU-X1) and 6 solid-state lasers for confocal imaging in real time. FRAP was achieved through the use of the photoconversion/photoactivation module. Areas of 1.5 μm^2^ area were bleached from at least six cells for each population were photobleached and analyzed and their fluorescence recovery was monitored and images were acquired with an EMCCD camera (Andor iXon ultra). Analysis of the intensity traces were carried out using the easyFRAP software (39) and corrected for photobleaching and normalized with pre-bleaching intensity.

### Bacterial expression vectors

A STOP codon was introduced to the pET28-Eg5 using a forward 5’CCTGAAGTGAATCAGAAATGAACCAAAAAAGCTTTGATTAAGG-3’ and a reverse 5’-CCTTAATCAAAGCTTTTTTGGTTCATTTCTGATTCACTTCAGG-3’ primers. Following sequence verification, the NcoI-HindIII insert of the pET28 Eg5 _(1-368)_ was ligated into the pETM11 NcoI-HindIII linearized vector. The pETM11 Eg5_(1-368)_ was then *in vitro* mutagenized by PCR using Phusion DNA Polymerase using the same primers as above. All resulting plasmids were sequence verified.

### Purification of pET11 Eg5 _(1-368)_ WT and mutants of Eg5 motor domain

BL21(DE3) cells carrying the pETM11 Eg5 _(1-368)_ WT and mutant Eg5 were induced for expression with IPTG (0.5mM) overnight at 18°C. Bacterial pellets were resuspended in cold Lysis Buffer (50mM Tris pH 6.8, 250mM KCl, 2mM MgCl_2_, 20mM imidazole) supplemented with 1⁄2 tablet of a mixture of protein inhibitors (Complete EDTA-free, Roche Applied Science), 2.5mg of lysozyme and 100µM phenylmethylsulfonyl fluoride (PMSF). Cells were lysed by sonication with 10 cycles of 30s of pulse and 60s of turn off at 50% amplitude in ice. Lysates were incubated with 1mM of DNAse and 10mM of MgCl_2_ during 30 min at 4°C to digest the DNA, centrifuged at 4°C for 30 min at 15000 rpm and the supernatants were collected and filtered (diameter of the filter = 0.2um) before being loaded onto a HisTrap HP 5mL column (GE Healthcare), equilibrated (using an AKTA design FPLC system) with buffer A (50mM Tris at pH 6.8, 250mM KCl, 20mM imidazole and 2mM MgCl_2_). The column was washed with 20 column volumes with buffer A. Finally, a gradient elution was performed over ten column volumes of buffer B (50mM Tris pH 6.8, 250mM KCl, 500mM imidazole, 2mM MgCl_2_) and 1.5mL fractions were collected. Based on the chromatogram fractions the interesting fractions were selected and run on 10% SDS-PAGE electrophoresis. Fractions containing the Eg5 motor domain were pooled together and then digested with 6-His-TEV protease, previously purified in the laboratory (1mg TEV/20 mg of substrate). Digestion was carried out for 1 hr at room temperature and then the protein solution was dialyzed overnight with 1L of Dialysis Buffer (50mM Pipes, pH7.3,50 mMNaCl), using a 10kDa MW cut-off dialysis membrane. TEV cleavage was confirmed by SDS-PAGE electrophoresis and the uncleaved motor as well as the His-TEV was removed by a second purification on HisTrap HP 5mL column. The cleaved motor was recovered from the HisTrap unbound fraction and was concentrated using spin concentrators (10kDa MW cut-off; Millipore). Protein samples were concentrated and frozen in small aliquots in liquid nitrogen and kept frozen at -80°C. Protein concentration was determined using the Bradford reagent at Abs_595_ using BSA as a protein standard.

### Mant-ADP release assay par fluorescence

Bacterially expressed and then isolated motor domain of Eg5(WT) and Eg5(T107N) (1.5μM final) were injected in the cell compartment of the fluorimeter Bio-Logic MOS 250 that contained Mant-ADP (10μM final) and FRET was detected by measuring fluorescence emission at 395nm after excitation at 285nm. STLC (25μM final) was injected into the cell and Mant-ADP was chased with excess ATP (1mM final). Alternatively, Eg5(WT) and Eg5(T107N) motor domains (1.9um final) were incubated with Mant-ADP (10μM final) and STLC (18μM final) in 10mM MgCl_2_, 6% DMSO (control) and the loss of FRET following addition of excess ATP (1.2mM final) was measured in black 384-well plates (Greiner ref. 781076, Greiner Bio-One) on a Clariostar plate reader (BMG Labtech) using a 285nm filter excitation at 285nm filter and 395nm emission filter and a LP565 dichroic mirror.

### Basal and microtubule stimulated ATPase assays

The enzymatic activity of kinesin Eg5 were carried out using the pyruvate kinase (PK)-lactate dehydrogenase (LDH) coupled assay in buffer A25 (40, 41). Experiments were performed in ATPase buffer A25 (25mM ACES/KOH (pH 6.9), 2mM magnesium acetate, 2mM potassium EGTA (SIGMA) (pH 8), 0.1mM potassium EDTA (SIGMA) (pH 8) and 1mM β-Mercaptoethanol) supplemented with 2mM PEP (SIGMA), 0.25mM NADH (SIGMA), 17.4 g/mL LDH (SIGMA) and 34ug/mL PK (SIGMA). Motor, microtubules, and ATP concentrations used were optimized. Kinetics were monitored in a TECAN-SUNRISE photometer using a 96-well plate (Greiner) by measuring the loss of NADH at OD_340_. *K_cat_* rates were determined from the change in OD_340_ per second after steady state has been reached, typically after a few minutes. All experiments were performed in triplicate at room temperature. Data were analysed using GraphPad Prism 7.

## Results

### Isolation of STLC-resistant cell lines

**Figure 1:**
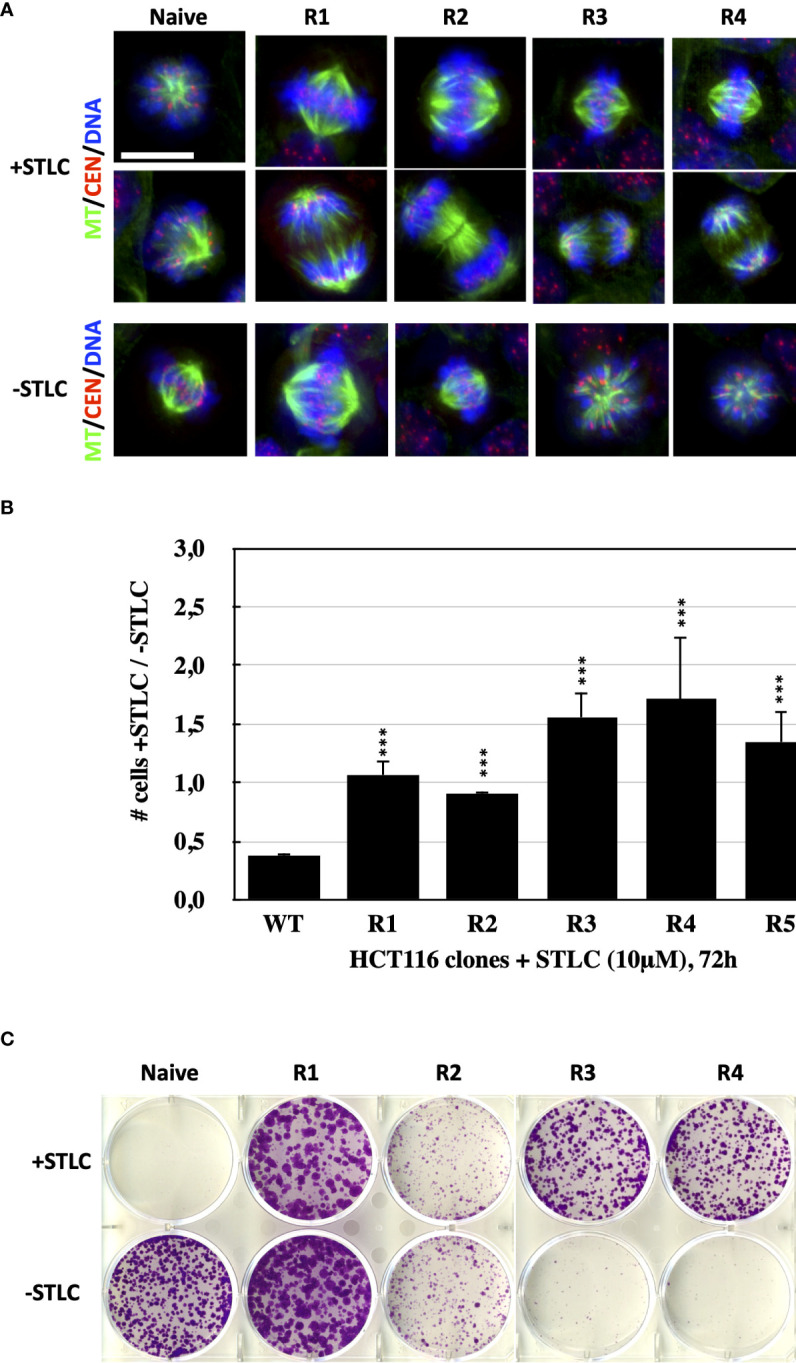
Bipolar spindle formation and proliferation of STLC-resistant cancer cells in the presence of STLC. **(A)** Immunofluorescence microscopy images of mitotic cells from naive and selected STLC resistant cells in the presence STLC 10 μM (upper two rows) and in the absence of STLC (lower row of images). Spindle microtubules were detected with an anti-tubulin antibody (green); centromeres with an auto-immune antibody (red) and chromatin with DAPI (blue). In contrast to the unselected naive cells, all resistant clones appeared to have normal bipolar spindles and were capable of proceeding to anaphase. STLC resistant clones R3, R4 and R5 in the absence of STLC had monopolar spindles. **(B)** Proliferations assays with the selected clones in the presence and in the absence of STLC. Ratio of the number of cells in the presence of STLC divided by the number of cells in the absence of inhibitor for unselected HCT116 cells and cells from the STLC resistant clones R1, R2, R3, R4 and R5, after 72h. Asterisks indicate significance values; *** p < 0,0001. **(C)** Crystal violet-stained colonies of parental HCT116 cells and drug-resistant lines (resistant clones R1, R2, R3, R4 and R5) after 14 days of exposure to STLC (upper panel) and following wash-out of the STLC (lower panel).

To determine whether human cancer cells can develop resistance to Eg5 inhibitors, we treated HCT116 cells with a cytotoxic concentration of STLC, a selective Eg5 inhibitor (31). HCT116 cells offer the advantage since they are defective in their DNA mismatch repair pathway that are hypermutagenic (42) and they might contain larger numbers of variants for selection under the conditions of our selection. In addition, HCT116 cells have no detectable P-glycoprotein (43), reducing the possibility of resistance due to induction of drug pumps. When ∼5 × 10^6^ cells were continuously exposed for 4 weeks at 10 μM STLC, colonies appeared from which following ring selection, we generated 5 cell lines, designated R1 through R5. Detection of spindle formation in the STLC-resistant clones by indirect immunofluorescence microscopy revealed that indeed the cells were capable of forming bipolar spindles in the continuous presence of the Eg5 inhibitor and cells in various mitotic phases, notably anaphases, were present (**Figure 1A**). Cells from the selected clones were then subjected in proliferation assays for 72h and the ratio of number of cells in the presence of STLC over the number of cells in the absence of the inhibitor was determined from each clone (**Figure 1B**). The calculated ratio from the unselected, naive wild-type HCT166 cells was as expected lower than one (0,38 ± 0,008) since cells stopped to proliferate due to a mitotic block imposed by the spindle assembly checkpoint. However, the ratios for the cells of clones R1 and R2 were significantly higher compared to the wild-type and were close to one- or very close to one-, indicating that the presence of the inhibitor did not interfere with cell proliferation. Interestingly, the ratios for the cells from clones R3, R4 and R5 were significantly higher than one, indicating that in the absence of the inhibitor the cells of each of the clones do not proliferate. The low proliferation of the cells from clones R3, R4 and R5 in the absence of STLC coincides with the inability of the cells to form bipolar spindles in the absence of the inhibitor (**Figure 1A**). The monopolar spindles in the cells of clones R3, R4 and R5 in culture media without STLC resemble the monopolar spindles of cells treated with Eg5 inhibitors. In accordance with the above observations, ∼1000 starting cells from the five clones, readily formed colonies at 10uM STLC after 14 days, whereas the naive unselected cells were potently inhibited; cells from clones R3, R4 and R5, were also not capable of growing in the absence of STLC (**Figure 1C**).

### Eg5 presence is required for bipolar spindle formation in the STLC resistant cells

**Figure 2:**
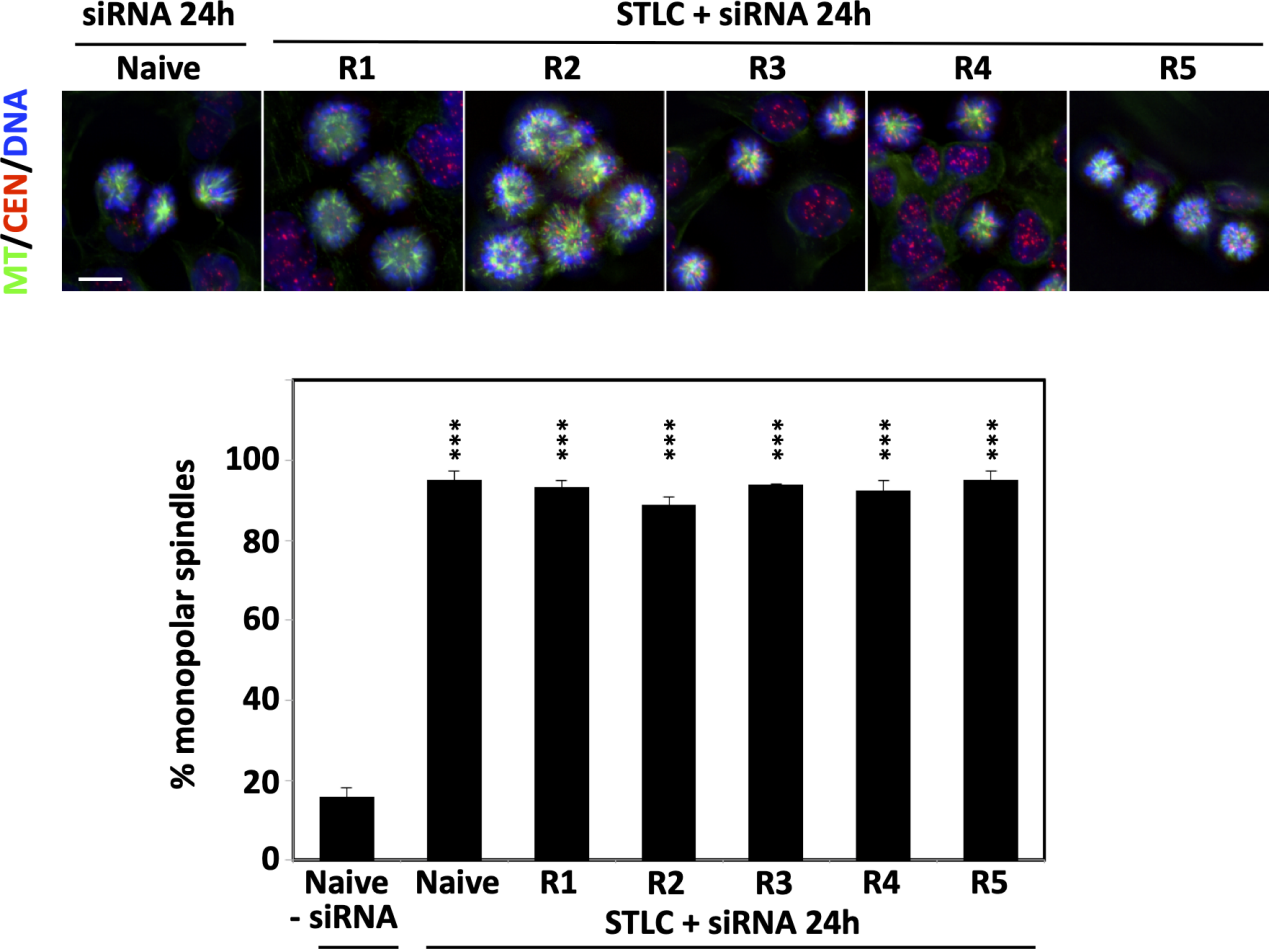
STLC resistant clones require the presence of Eg5 for growth in the presence of STLC. Immunofluorescence microscopy images of mitotic cells from unselected naive HCT116 cells and selected HCT116 STLC-resistant cells that were subjected to Eg5 siRNA mediated depletion of Eg5. Spindle microtubules were detected with an anti-tubulin antibody (green), centromeres with an auto-immune antibody (red) and chromatin with DAPI (blue). Scale bar in the images corresponds to 10 μm. The % of monopolar spindles following RNAi mediated Eg5 depletion in the presence of STLC was calculated for each the cell lines. Asterisks indicate significance values; *** p < 0,0001.

We then asked the question whether or not the formation of bipolar spindles in the STLC-resistant cells was still dependent on the presence of Eg5. We therefore treated the resistant cells with siRNA targeting Eg5 expression. Phenotypic analysis, by monitoring spindle assembly in the siRNA treated cells in the continuous presence of the STLC, revealed that the cells depleted of Eg5 were not capable of forming bipolar spindles as in all clones more than 90% of the spindles were monopolar, as they were in the depleted naive and untreated cells (**Figure 2**). Therefore, the data suggested that in the STLC-resistant cells the presence of Eg5 is necessary for the formation of spindles even in the presence of the inhibitor.

### Differential resistance to Eg5 inhibitors of STLC-resistant clones

**Figure 3:**
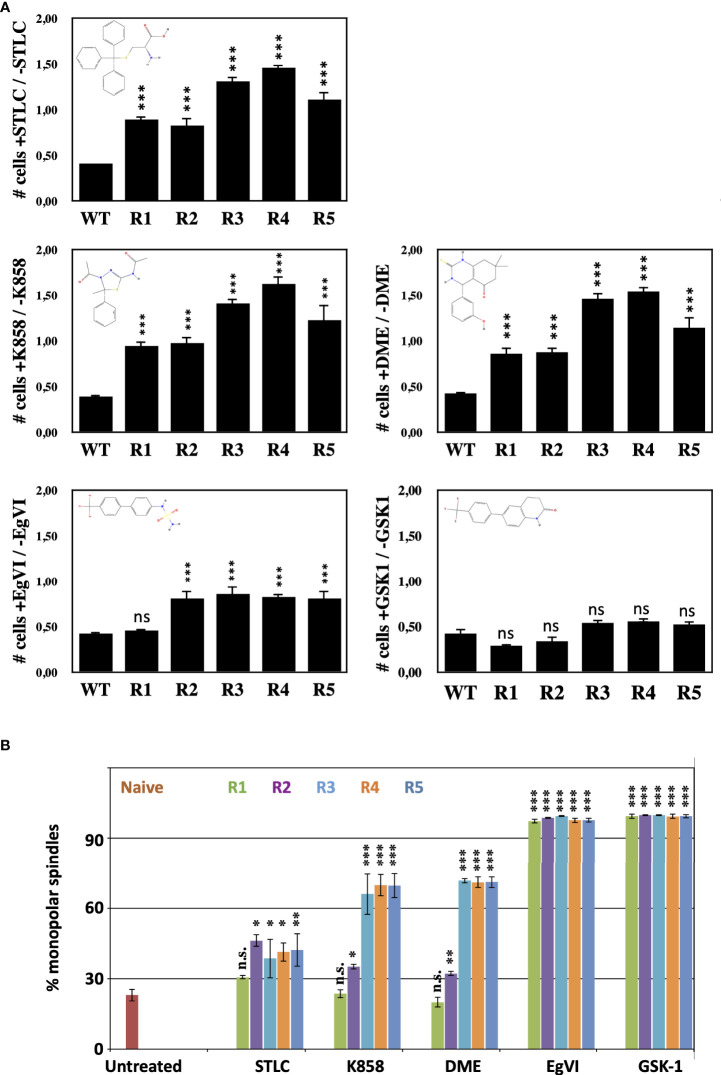
STLC resistant clones exhibit variable resitance to other Eg5 inhibitors **(A)** Proliferations assays with the selected clones in the presence and in the absence of STLC. Ratio of the number of cells in the presence of STLC (10μM) or K858 (7.5μM) or DME (5μM) or EgVI (5μM) or GSK-1 (0.5μM) divided by the number of cells in the absence of inhibitor for unselected HCT116 cells and cells from the STLC resistant clones R1, R2, R3, R4 and R5, after 72h. **(B)** The % of monopolar spindles in the presence of the different Eg5 inhibitors was calculated for each the cell lines from immunofluorescence microscopy images of mitotic cells (**Supplementary Figure S1**). Asterisks indicate significance values; ns – non-significant; * p < 0,01, **p < 0,001, *** p < 0,0001.

Since STLC belongs to the loop L5 binding class of Eg5 inhibitors, we tested if the STLC-resistant cell lines are also resistant to other loop L5 inhibitors with different chemical scaffolds such as K858 (44) and dimethylenastron (DME) (45), and to non-loop L5 inhibitors (ATP competitive inhibitors) EgVI and GSK-1 (32). Cells from the unselected wild type and the five selected STLC HCT116 resistant clones were analyzed in the presence and in the absence of each of the inhibitor in proliferation assays (**Figure 3A**) and the phenotype of the treated cells were analyzed by immuno-fluorescense microscopy (**Figure 3B**; **Figure S1A**). Following 72h exposure of the cells in the presence of the different Eg5 inhibitors, cells from all resistant clones were capable of proliferating in the presence of STLC, K858 and DME in contrast to the control whereas all cells were sensitive to the ATP competitive Eg5 inhibitors EgVI and GSK1. Microscopic analysis of the spindle phenotype of the treated cells revealed that for clones R1 and R2 the majority of the spindles were bipolar whereas in cells of clones R3, R4, and R5 were monopolar in the presence of the K858 and DME (**Figure 3B**; **Figure S1A**). All cells, however, were sensitive to the Eg5 ATP competitive inhibitors EgVI and GSK-1 (**Figures 3A, B**). We have also assessed the ability of the resistant clones their ability to form cell colonies by carrying out long term colony formation assays in the presence of the inhibitors (**Figure S1B**). In accordance to the 72h proliferation assays and mitotic spindle formation capability of the resistant cells, 21 days exposure of cells in the different Eg5 inhibitors showed in contrast to the wild type cells, cells from clones R1 and R2 were capable of colony formation and cells from clones R3, R4 and R5 to a limited extend. However, all cells were not able to form colonies in the presence of the ATP competitive Eg5 inhibitors EgVI and GSK1. The results suggest that all STLC resistant clones are sensitive to ATP competitive inhibitors and there is a variable resistance to the other loop L5 binding inhibitors, such as K858 and DME.

### Failure in mitosis in the absence of STLC in the STLC resistant and dependent cells

**Figure 4:**
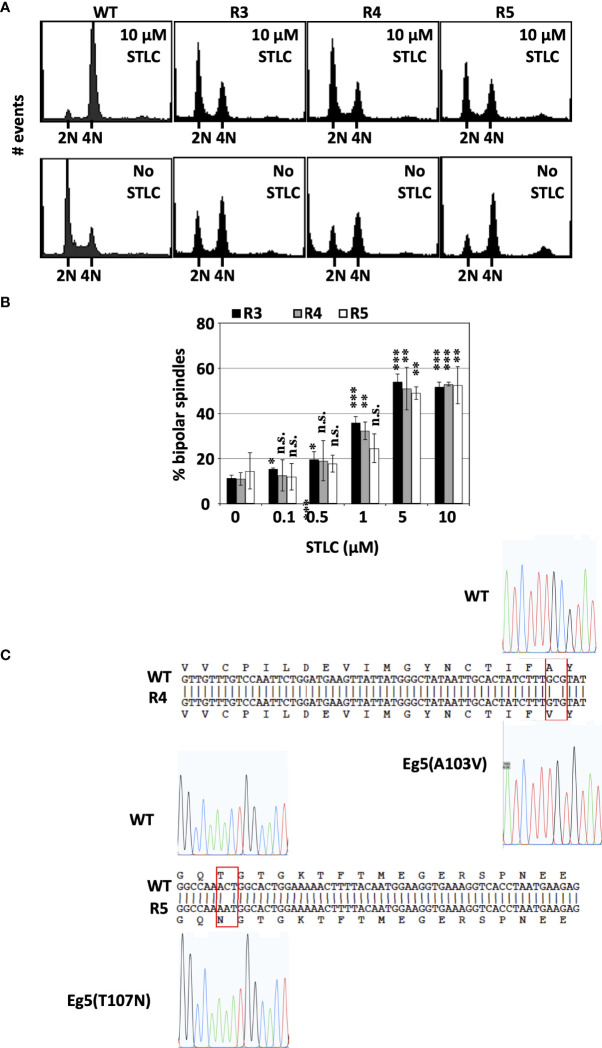
STLC resistance and dependence is linked to expression of mutant Eg5 **(A)** Cell-cycle profiles of unselected wild type HCT116 cells and STLC-resistant and -dependent clones, R3, R4 and R5 exposed to STLC (10 μM) (upper panels) and 24h following wash-out of STLC (lower panels). **(B)** The % of bipolar spindles in cell lines R3, R4 and R5 increase with increasing STLC concentration. Asterisks indicate significance values; ns – non-significant; * p < 0,01, **± p < 0,001, *** p< 0,0001. **(C)** DNA sequences of Eg5 (KIF11) cDNAs in WT and STLC-resistant and -dependent clones, R4 and R5 and the identified corresponding missense mutations. Clone R3 expressed the same T107N mutant Eg5 as the one identified in clone R5.

The cell cycle profile of the three cell clones that appeared to be STLC resistant and dependent were analyzed by flow cytometry and the ability of the cells to form bipolar spindles in a STLC concentration dependent manner. Cells from clones R3, R4, and R5 had a normal cell cycle profile in the presence of 10 μM STLC after 72h comparable to the naive untreated cells. However, in the absence of STLC as early as 24h after washing out the inhibitor, cells were enriched in G2/M (**Figure 4A**).

The enrichment of cells in G2/M in the R3, R4, R5 HCT116-clones in the absence of STLC was correlated with the presence of aberrant monopolar spindles (**Figure 4B**). There was an inverse correlation between the presence of bipolar spindles and decreasing concentrations of the inhibitor. In the absence and submicromolar concentrations of STLC the majority of the spindles were monopolar whereas at 5 and 10μM STLC more than 50% of the spindles were bipolar. The presence of bipolar spindles in the presence of the STLC offers an explanation why the cells continue to proliferate in the presence of the inhibitor and the lack of bipolar spindles in the absence of the inhibitor explain the dependency of the cells on the inhibitor.

### Identification of Eg5 Mutations in the STLC resistant and dependent cells

We then asked whether the drug-resistance in drug-dependent cell lines might be due to mutations in Kif11, the gene coding for Eg5 motor protein. Sequencing Kif11 cDNAs from the drug-resistant clones revealed that all 3 STLC-dependent lines carried point mutations in Kif11, yielding 2 amino acid substitutions, namely cells in clone R4 expressed Eg5(A103V) and clones R3 and R5 expressed Eg5(T107N) (**Figure 4C**). Therefore, we focused our attention to the Eg5(T107N) STLC resistant and dependent cell line since the mutation A103V was identified independently in a previous study (46) and was not further pursued.

### Resistance and dependence to clinical relevant Eg5 inhibitors

**Figure 5:**
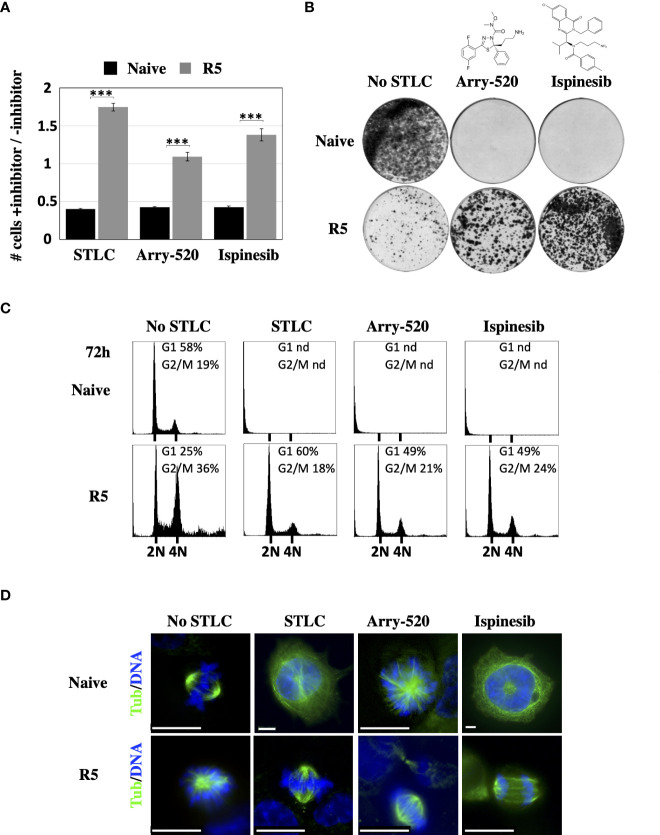
Response of the Eg5(T107) expressing cells to Arry-520 and ispinesib **(A)** Proliferation of STLC resistant and dependent cells in the presence and in the absence of STLC (10μM) or Arry-520 (10nM) or ispinesib (10nM). Ratio of the number of cells in the presence of STLC (10μM) or Arry-520 (10nM) or ispinesib (10nM) divided by the number of cells in the absence of the inhibitor for unselected HCT116 cells and cells from the STLC resistant clone R5, after 72h. Asterisks indicate significance values *** p < 0,0001;**(B)** Crystal violet-stained colonies of parental HCT116 cells and STLC-resistant and resistant clone R5 cells after 14 days exposed to either media or Eg5 inhibitors, Arry-520 (10nM) and ispinesib (10nM). **(C)** Cell-cycle progression of STLC-resistant and -dependent clone R5 exposed to STLC, Arry-520 and ispinesib for 72h. **(D)** Immunofluorescence microscopy images of mitotic cells from naive wild type (WT) and STLC -resistant and -dependent cell line clone R5, in the absence and in the presence of STLC, Arry-520 and ispinesib. Microtubules were detected with an anti-tubulin antibody (green) and chromatin with DAPI (blue). Scale bar in the images corresponds to 10 μm.

Due to the interesting STLC-resistance and at the same time STLC-dependent phenotype of the Eg5(T107N) expressing cells, we tested if they were also resistant and dependent to the clinical relevant Eg5 inhibitors, Arry-520 (47) and ispinesib (48), two inhibitors that entered clinical phase II clinical trials. The Eg5(T107N) expressing cells of HCT116 clone R5 were able to proliferate (**Figure 5A**) and in long term colony formation assay to form colonies (**Figure 5B**) in contrast to the naive HCT116 cells that they were not able to neither proliferate nor form colonies in the presence of either Arry-520 or ispinesib. In agreement with the colony formation data, flow cytometric analysis of the treated cells after 72h showed normal cell cycle profiles in the presence of either Arry-520 or ispinesib as they did in the presence of STLC, whereas naive cells exposed to the either of the three inhibitors were absent since they could not proliferate (**Figure 5C**). Indirect immunofluorescence analysis of the treated cells also showed that the Eg5(T107N) expressing cells exhibited normal bipolar spindles and/or normal anaphase spindles in the presence of either of the three inhibitors whereas the naive cells after 72h were in either in interphase with an aberrant large nucleus, prominent centrosome, nucleated microtubules and/or monopolar spindles (**Figure 5D**).

### Ectopic expression of Eg5(T107N) mutant restores bipolar spindle formation in the presence of STLC

**Figure 6:**
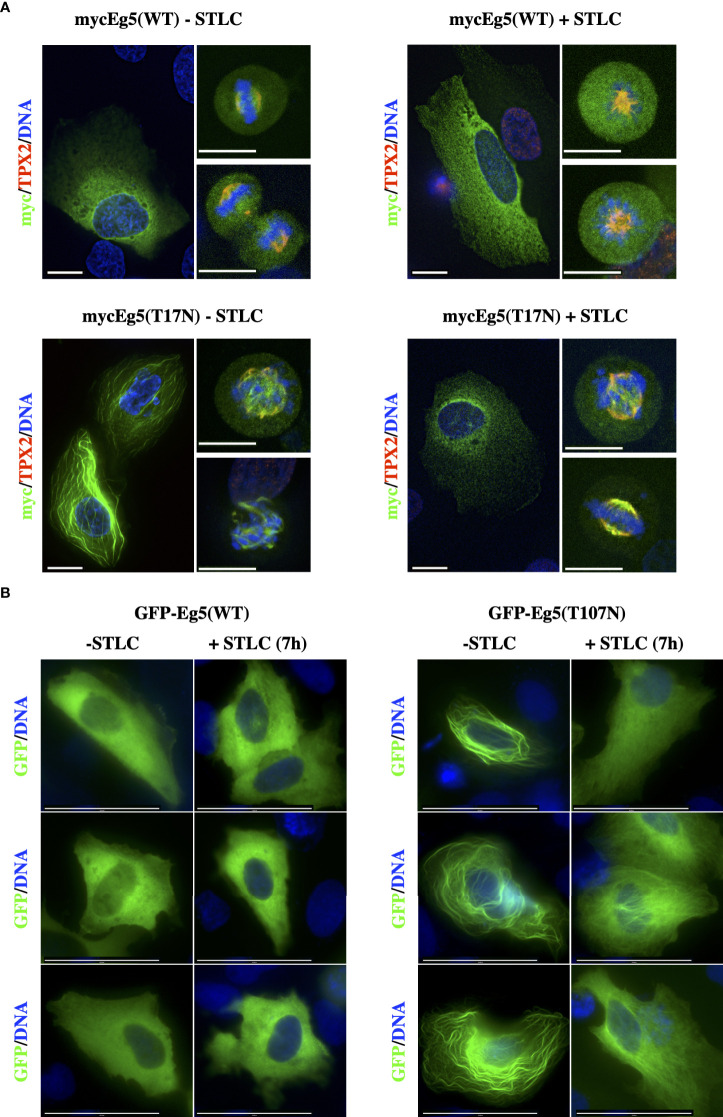
STLC resistant Eg5(T107N) variant binds and bundles microtubules in the absence of STLC. **(A)** Myc-tagged Eg5(WT) and -Eg5(T107N) were transfected for expression in U-2OS cells. Immunofluorescence microscopy images of cells untreated or in the presence of STLC (10μM) 24h post transfection fixed and stained; Eg5 was detected by an anti-myc (green) and mitotic spindle microtubules were detected by an anti-TPX2 (red) and chromatin by DAPI (blue). **(B)** GFP-tagged Eg5(WT) and -Eg5(T107N) were transfected for expression in U-2OS cells for 24h and fixed and stained for indirect immunofluorescence microscopy. Scale bar corresponds to 10 μm.

To test whether the Eg5(T107N) mutant is sufficient to cause drug resistance, we ectopically expressed as Myc-tagged either the wild type Eg5(WT) or the mutant Eg5(T107N) in U2-OS cells (**Figure 6A**). The Myc-Eg5(WT) either in the absence or the presence of STLC appeared to be diffused in the cytoplasm and in mitotic cells there was some decoration of the spindle particularly in the spindle poles (**Figure 6A** upper panels). Strikingly, the Myc-tagged Eg5(T107N) in the absence of STLC appeared to exhibit a strong microtubule bundling activity in interphase cells; in mitotic cells there were also bundled microtubules and no bipolar spindles were present (**Figure 6A**; lower left panels). In marked contrast, in the STLC treated cells, expression of Myc-Eg5(T107N) in interphase showed no microtubule bundles and in mitotic cells the spindles were bipolar (**Figure 6A**; lower right panels). The obtained data offer an explanation why the cells in STLC dependent and resistant clone R5 are capable of proliferating in the presence of STLC and not in the absence of STLC.

Similar strong microtubule bundling after expression of Eg5(T107N) has been previously reported in cells expressing two other Eg5 mutants, Eg5(G268V) (49) and Eg5(T112N) (50). The microtubule bundling activity of Eg5(G268V) was previously attributed to the fact that since the mutation is in the switch II ATP binding domain it drives the motor to be at its rigor state and therefore leading the four motor domains of the Eg5 tetramer to bind tightly to the microtubules in a non-exchangeable manner and forcing the microtubules to bundle. Since the Thr107 amino acid is located in the P-loop of the ATP binding site we tested if the mutant T107N, like the G268V, drives the motor to bind in a non-exchangeable manner to microtubules. First we constructed GFP-Eg5(WT) and GFP-Eg5(T107N) mammalian expression vectors and determined if the GFP chimeras behave the same as the Myc-tagged Eg5. Similar to Myc-tagged constructs the GFP-Eg5(WT) was distributed in a diffuse manner in the cytoplasm of interphase cells and the GFP-Eg5(T107N) induce strong interphase bundles (**Figure 6B**).

**Figure 7:**
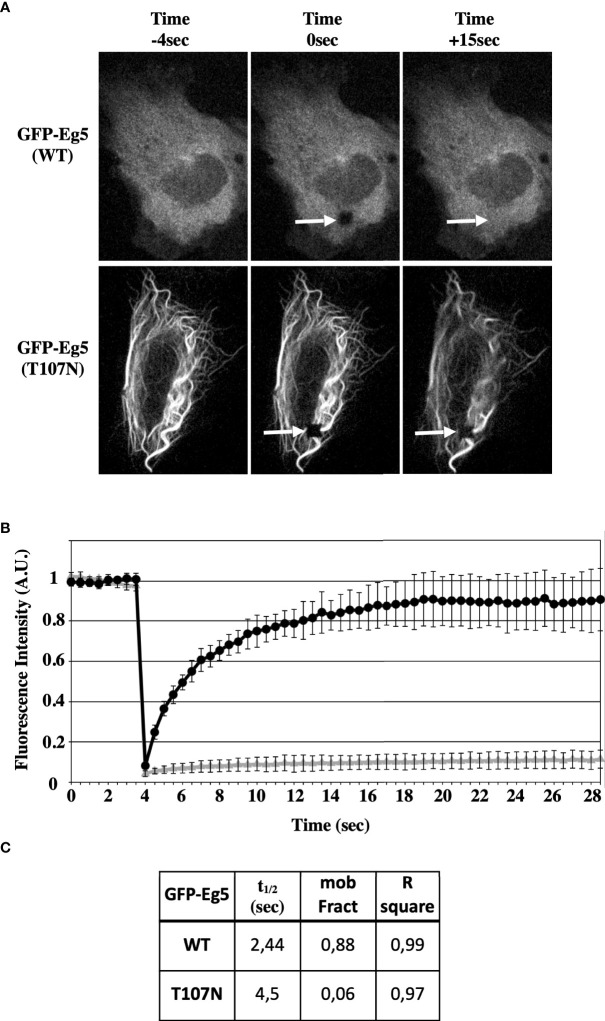
STLC resistant Eg5(T107N) variant binds microtubules in a non-reversible manner in the absence of STLC. **(A)** GFP-tagged Eg5(WT) and -Eg5(T107N) were transfected for expression in U-2OS cells. Time lapse video microscopy images 48h following transfection before (-4 sec), immediately after FRAP (0 sec) and after FRAP (15 sec) are shown. Arrows indicate the site of the photobleaching. **(B)** Recovery of the fluorescence intensity after FRAP. **(C)** The measured mobile fraction and the half time represent the ability of GFP-Eg5 to actually replace the photobleached in cytoplasm of living cell during interphase.

Then, the dynamics of the microtubule binding properties of the GFP-Eg5(T107N) were compared with those of GFP-Eg5(WT) by FRAP analysis in living U2-OS cells. In GFP-Eg5(WT) expressing cells, where the distribution of GFP-Eg5 appears as diffused in the cytoplasm similar to endogenous Eg5, following bleaching in the region of interest (ROI), the fluorescent signal gradually increased during the recovery phase (25s) because of the inflow of unbleached GFP-Eg5 into the bleached area (**Figure 7A**). In contrast to the WT, the GFP-Eg5(T107N) decorated strongly microtubule bundles in the cytoplasm of interphase cells (**Figure 7A**). The GFP-Eg5(T107N) exhibited almost no recovery (mobility fraction = 0,06) in the bleached ROI confirming the strong almost irreversible binding of the rigor mutant of Eg5 to microtubules at the time scale of the observation (**Figures 7B, C**). Therefore, the FRAP data suggest that the microtubule binding and bundling observed in GFP-Eg5(T107N) expressing cells in the absence of the inhibitor is probably due to higher microtubule affinity properties of the mutant Eg5 compared to the control.

### *In vitro* Activity of Eg5(T107N) mutant

**Figure 8:**
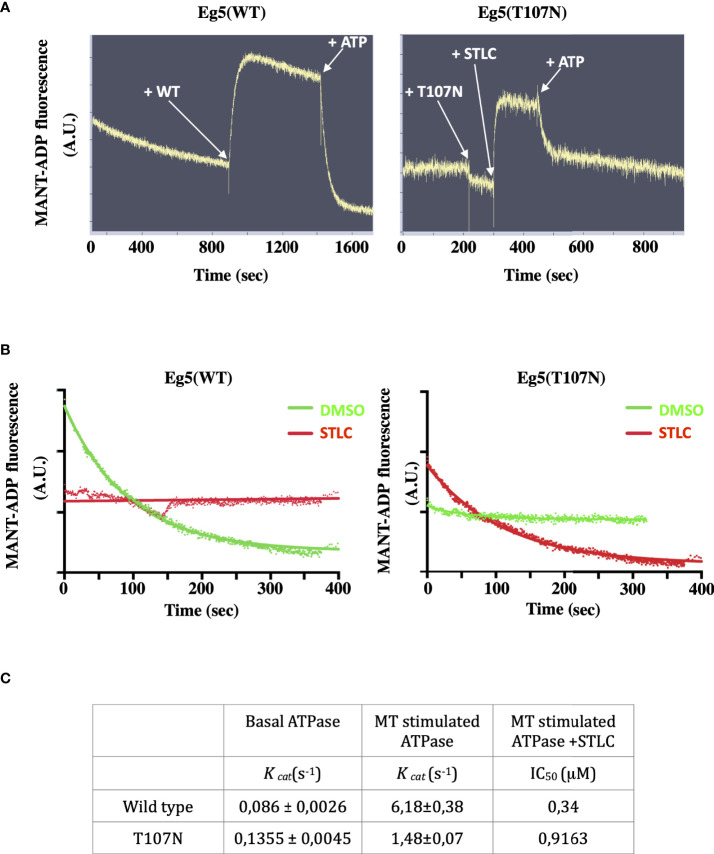
STLC dependent binding of ATP in the nucleotide pocket of Eg5(T107N) motor domain. **(A)** FRET of Mant-ADP is detected upon addition of the Eg5(WT) motor domain and is lost after been chased by the addition of cold ATP. In contrast, FRET of Mant-ADP is not detected upon addition of the Eg5(T107N) domain unless STLC is added. **(B)** Nucleotide exchange in the Eg5-STLC complex. Once STLC is bound to the Eg5(WT) pre-incubated with Mant-ADP the nucleotide is not exchangeable. Pre-incubation with DMSO allows the chase of Mant-ADP by ATP. In contrast, there is an exchange of the nucleotide in the Eg5(T107N)-STLC complex but not in the in the absence of STLC (DMSO control). **(C)** Kinetic parameters of the basal ATPAse activity of Eg5(WT) and Eg5(T107N) and the microtubule stimulated ATPase in the presence or the absence of STLC.

Previous studies have suggested that Eg5 inhibitors can be divided into two classes, the loop-L5 type of inhibitors that actually promote weak binding of the motor to microtubules (15, 31, 51, 52), and the rigor-like inhibitors that are ATP competitive inhibitors and induce Eg5 strong microtubule-binding state (32, 53, 54). Furthermore, the loop L5 binding inhibitors are able to allosterically inhibit the exchange of the bound ADP with ATP and therefore the ATPase activity of the motor is inhibited and the motor cannot efficiently engage with the microtubules. The ternary Eg5-ADP-STLC complex might be able to bind MTs in a low-friction mode without productive ATP hydrolysis and coupled conformational changes. The mutant Eg5(T107N) behaves like a motor that is in rigor state and that in the presence of the STLC the motor is able to complete the chemomechanical step. In order to test the ability of the motor domain to bind and exchange the nucleotide in the ATP binding pocket in the absence and in the presence of STLC we determined the ability of the motor domain to bind a fluorescent analogue of ATP, Mant-ADP (55). Addition of the wild type motor domain in a Mant-ADP containing solution leads to an exchange of the motor bound ADP with Mant-ADP and with a resulting increase in fluorescence (excited at 285 nm) due to FRET from the tryptophan and tyrosine of Eg5 (**Figure 8A**, left panel). However, when the Eg5(T107N) motor domain was added to Mant-ADP there was no increase in FRET detected unless STLC was added (**Figure 7A**, right panel). Bound Mant-ATP could be then chased out by excess amount of ATP in both the wild type and the mutant. The data show that in the absence of microtubules the nucleotide pocket of Eg5(T107N) is in a state that cannot be accessed by a free nucleotide unless there is some allosteric conformations that are transmitted to the nucleotide binding pocket following binding of STLC to the loop L5. Binding of the inhibitor opens the nucleotide binding pocket allowing the entry of the nucleotide.

We then asked if in the continuous presence of STLC bound to the motor domain the bound Mant-ADP can be chased out by cold ATP. As expected for the wild type, in the presence of STLC there was no nucleotide exchange whereas the Mant-ATP was readily exchanged if the motor was pre-incubated with DMSO instead of STLC (**Figure 8B** left panel). However, in the case of the Eg5(T107N) motor domain pre-incubated with Mant-ADP in the presence or absence of STLC the Mant-ADP could be chased out by ATP only when STLC was present (**Figure 8A** right panel). The results suggest that in the Eg5(T107N), in contrast to the wild type-STLC complex, binding of STLC to loop-L5 permits the exchange of the bound ADP by ATP. Therefore, the next question we addressed was whether the mutant Eg5(T107N) can hydrolyze ATP in the presence of STLC.

The motor domain of the wild type Eg5, like all other kinesin motor proteins, is known to possess a basal ATPase activity (56) which is greatly stimulated in the presence of microtubules. We therefore measured the microtubule stimulated ATPase activity of wild type and that of Eg5(T107N) motor domains *in vitro 5* (**Figures 2SA, B**). The mutant displayed similar a *k_cat_* value for basal ATPase activity (0,63fold increase) compared to the wild type Eg5 (**Figure 8C**). Furthermore, there was only a 10.9x increase in *k_cat_* value for the MT stimulated ATPase activity compared to the 71.8fold increase for the wild type. Although both wild type and mutant showed a dose response loss of activity in the presence of increasing concentrations of STLC, there was a 2,7fold increase in the IC_50_ for the mutant compared to the wild type (**Figure 8C**; **Figures S2C, D**). Therefore, the lower sensitivity (higher IC_50_) of the mutant to STLC, coupled with the ability of the mutant to exchange the nucleotide only in the presence of the inhibitor in contrast to the wild type, offers a likely explanation why the mutant Eg5 motor is still active in cells in the presence of STLC.

## Discussion

Eg5 is a valid target for the development of novel generation of specific anti-mitotic targets as cancer chemotherapeutic agents with less secondary effects compared to those linked to microtubule targeting agents (57). One hindering caveat of selective inhibitors to a given target is the emergence of subclones of tumour cells, preexisting or not, having mutations in the target, rendering the target resistant (58) (59). Previous data from our group and others have shown that indeed certain mutations in the helix α2/loop L5/helix α3 allosteric binding site of Eg5 can confer resistance to loop L5 inhibitors (26, 28, 38, 60).

In this report following the selection of HCT116 cells in the presence of the Eg5 inhibitor STLC, we have isolated five STLC resistant clones, arbitrarily named as R1, R2, R3, R4, and R5, that proliferate in the presence of the inhibitor at concentrations that block cellular growth due to an imposed mitotic block. Interestingly, three of the identified resistant clones were also STLC-dependent, the cells could not proliferate in the absence of STLC. The failure of cell proliferation of the STLC -resistant and -dependent cells was attributed to that the cells in the absence of STLC could not build bipolar spindles and therefore remained blocked in mitosis with the characteristic monopolar spindles, a phenotype widely observed when the Eg5 activity is missing either due to enzymatic inhibition or absence due to depletion by RNAi. Therefore, the STLC-resistance and dependency on Eg5 could most likely be attributed to the intrinsic properties of the Eg5 motor expressed in the cells.

Sequencing of the Eg5 cDNAs isolated from the three STLC-resistant and -dependent clones, raised in this report, identified two amino acid substitutions at positions, A103V and T107N that could be possible candidates for the observed resistance. The failure to detect Eg5 mutations in the two other clones may be related to the fact that the primers used for RT-PCR amplification covered only the part of the motor domain that included the helix α2/loop L5/helix α3 allosteric binding site of Eg5 open reading frame and not all the motor domain of Eg5. An additional contributing factor might be the functional plasticity of mitotic kinesins substituting for loss of Eg5 function (29, 30, 49).

In the absence of STLC, expression of the Eg5(T107N) mutant in cells led to strong microtubule cross linking in interphase and mitotic cells leading to mitotic failure. However, in the presence of STLC the mutant Eg5 had a diffused intracellular distribution resembling that of the wild type protein. FRAP assays in STLC devoid Eg5(T107N) expressing cells showed that the mutant protein is bound to microtubules in a non-exchangeable manner suggesting that the mutant motor is bound most likely in its rigor state. Since Eg5 is a tetramer, composed of two antiparallel dimers (4), the rigor state of the mutant would most definitely lead to a tight crosslinking of microtubules. Previous screening for resistance to STLC have led to the identification of Eg5(G268V) as being also responsible for the observed STLC resistance (49). The Eg5 residue G268 is located in the switch II nucleotide binding area and like the T107N mutant, the G268V traps the motor in a microtubule binding state leading to a strong microtubule crosslinking. Therefore, both mutants convert Eg5 from a motile force generator to a static microtubule crosslinker. Interestingly both resistant clones, G268V and T107N, appear to be dependent on the presence of the motor since the cells of both clones depleted of the Eg5 by siRNA do not proliferate. In the case of Eg5(G268V) expressing cells, acquired resistance was linked to the functional plasticity of mitotic kinesins, like KIF15, substituting for loss of Eg5 wild type function. KIF15 is a dimeric kinesin and through its two N-terminal motors and two C-terminal nonmotor MT-binding sites, is able to crosslink and slide MTs (61). In the G268V resistant clone, the mitotic kinesin KIF15, which is normally restricted to kinetochore-MTs because of its preferential binding to MT bundles, in the presence of Eg5(G268V) induced crosslinking, changes its distribution and is enriched in the rest of spindle microtubules contributing to the formation of normal bipolar spindle (49). However, in the case of Eg5(T107N) expressing cells, microtubule crosslinking is present only in cells that are devoid of STLC. Therefore, the Eg5(T107N) acquired resistance appears not to be dependent on the plasticity of mitotic kinesins substituting for loss of Eg5 function. It appears to be due to intrinsic enzymatic properties of the mutant motor. Support for the need of Eg5 activity in the STLC-resistant and dependent cells is the fact that the resistant cells remain sensitive to Eg5 inhibition mediated by Eg5 inhibitors that are known to bind to a different binding site other than the helix α2/loop L5/helix α3 allosteric binding site and cause the inhibited motor domain to bind to microtubules.

The enzymatic data showed that the change of an invariant threonine (T) in the nucleotide-binding GQTGTGKT motif or P-loop of Eg5 by asparagine (N) is linked to cellular resistance to STLC, a loop L5 binding inhibitor. The presented nucleotide exchange data using Mant-ADP showed that nucleotide entry in the Eg5(T107N) is absolutely dependent on the binding of STLC in the inhibitor binding site, α2/loop5/α3 allosteric pocket, which is approximately 10 Å away from the ATP-binding site. In Eg5, P-loop Thr107 is involved in the positioning of the γ-phosphoryl oxygen of ATP (62). Furthermore, according to the two-water mechanism of hydrolysis of ATP by Eg5 (62), during the chemomechanical step there is an opening of the nucleotide pocket allowing the release of P_i_, which is coupled with the reorientation of the side chain of Thr107 allowing its binding to Glu270, as observed in the Eg5(WT)-ADP structure. The substitution of Thr107 with an amino acid with bulkier side chain, like Asn, may make the binding of the ATP less favorable and the reorientation of the P-loop impossible. However, the allosteric conformational changes imposed by the binding of STLC may open the nucleotide pocket and allow the entry of ATP leading to its hydrolysis and completing the necessary chemomechanical reaction leading to stepping of the motor.

It has been proposed that homotetrameric Eg5 crosslinks antiparallel MTs in spindles the same way as myosin crosslinks actin filaments in thick filaments (63). In both cases independent myosin or Eg5 motor heads can exert forces causing actin or microtubule filament sliding, respectively. In the presence of the Eg5(T107N), and in the absence of the inhibitor all the motor heads of the tetramer remain bound to the microtubules in a rigor state. The rigor state of the motor then is the cause of the observed strong microtubule bundling in the Eg5(T107N) expressing cells. In the presence of the inhibitor and because of the allosteric modifications transmitted into the nucleotide binding pocket of the motor domain, new ATP can enter in the nucleotide pocket releasing the motors from the microtubules, microtubules bundles are resolved and a new chemomechanical step of the four motor domains of the Eg5 tetramer can be initiated. However, how would Eg5(T107N) exert its motility functions in the presence of the inhibitor?. Firstly, because of the lower sensitivity of the mutant (2,7x higher IC_50_ for STLC compared to the wild type) even if only a few motor heads can complete ATP hydrolysis in the presence of STLC there will be enough to exert force-generating events necessary for directional microtubule sliding. Secondly, in addition to the ATP-consuming directional motion, Eg5 exhibits also a diffusive component not requiring ATP hydrolysis (64). This diffusional mode was also reported in the presence of Eg5 inhibitor monastrol (65), the prototype of the loop-L5 binding class of inhibitors (6) and it can also occur at physiological ionic strength (64). In the presence of the STLC that binds to the loop-L5 binding site as monastrol, following the exchange of the nucleotide in the Eg5(T107N) and the release from microtubules, the mutant motor can exhibit a diffusive mode of motility and in combination with its directional mode of motility, the tetrameric Eg5(T107N)-STLC complex will possess crosslinking and motile activity enough to contribute to the formation of a bipolar spindle. Therefore, the allosteric modifications that follow STLC binding are needed for the observed resistance to STLC in the Eg5(T107N) expressing cells.

The resistance dependent on the presence of the bound allosteric inhibitor reported here is an additional manifestation of resistance by allostery (66). Previous studies have shown that residue A133 is necessary for transmitting a perturbation pathway through the protein to the nucleotide-binding site when binding the inhibitor; when mutated to D133 the allosteric transmissions provoked by SB743921, another loop-L5 class of Eg5 binding inhibitors, are blocked conferring thus resistance. In the case of the Eg5(T107N) mutant, the perturbations that follow the binding of the inhibitor not only allow but they appear to be necessary for the exchange of the nucleotide in the ATP binding pocket, a step that is necessary for the completion of the ATP hydrolysis cycle and stepping of the motor (67).

It is interesting to also note that the STLC dependent cells remain sensitive to the class of inhibitors that are considered as ATP competitive and bind to a different site other than the STLC binding site. Furthermore, the resistance of the Eg5(T107N) cells appear not to be limited to STLC because the cells remain relatively insensitive to other loop L5 inhibitors that have entered clinical trails such as Arry-520 and ispinesib. However, the resistance and dependency it is not universal for all loop-L5 inhibitors since K858 and DME appear to inhibit growth in the STLC resistant and dependent cells. It is interesting to note that although DME has a different chemical scaffold compared to STLC or Arry-520 or ispinesib, K858 is a smaller, pre-optimised version of Arry-520 that lacks the key substituents necessary for potent inhibition. The results are consistent with our recent observation that subtle differences in ligand binding and flexibility in both compound and protein may alter allosteric transmission from the loop L5 site that do not necessarily result in reduced inhibitory activity in mutated Eg5 structures. Thus, it appears that resistance to Eg5 inhibition may be chemical scaffold dependent and suggest that a chemotherapeutic combination therapy employing different inhibitors targeting the same target may limit acquired resistance. More recently it was shown that cells expressing either Eg5(D130A) or Eg5(L214A) are resistant to STLC and to Arry-520, a phase II clinical candidate. In contrast, the Eg5(L214A) expressing cells were sensitive to ispinesib an inhibitor that share the same binding site as STLC and Arry-520 (20). The data allow us to predict that resistance and dependence by allostery to Eg5 inhibitors may also occur in cells and once it is diagnosed it may be beneficial since cessation of the chemotherapeutic regimen may be beneficial to the patients because drug dependent tumor cells will cease to proliferate. The data also suggest that combining allosteric and orthosteric Eg5 inhibitors like the ATP competitive type of Eg5 inhibitors may be a good strategy to circumvent drug resistance.

The presented data bring attention to how different inhibitors to the same target could provoke different responses to treatment (due to their different biochemical structure) and mode of action, and how, in the case of Eg5 kinesin, a mutation in the motor ATP binding site could possibly make the difference. Within the context of personalized and precision medicine, the data also point out the need of carefully considering that missense mutations in specific hotspots on the target may impair the function of some drugs but possibly not all of them. Therefore, our work highlights not only the importance of detecting mutations responsible for intrinsic resistance before even the beginning of the targeted therapy, but also the requirement to follow, through biopsy, the response to treatment in order to detect mutations that would confer an acquired resistance.

## Data availability statement

The original contributions presented in the study are included in the article/**Supplementary Materials**. Further inquiries can be directed to the corresponding author.

## Author contributions

R-LI carried out cell based assays and contributed in the construction and use of cDNAs. SD carried protein isolation and enzymatic characterization of the isolated motor proteins. IG-S contributed to the structural interpretation and analysis of the data. DS designed, supervised, and participated in the collection of the data, carried out microscopy and FRAP experiments and carried out analysis and interpretation of the entire presented data. All authors contributed to the article and approved the submitted version.

## Funding

This work used the platforms of the Grenoble Instruct-ERIC Center (ISBG: UMS 3518 CNRS-CEA-UGA-EMBL) with support from FRISBI (ANR-10-INSB-05-02) and GRAL (ANR-10-LABX-49-01) within the Grenoble Partnership for Structural Biology (PSB). Part of this work was supported by La Ligue Contre Le Cancer Comité du Rhône.

## Acknowledgments

This work used the platforms of the Grenoble Instruct-ERIC Center (ISBG: UMS 3518 CNRS-CEA-UGA-EMBL) with support from FRISBI (ANR-10-INSB-05-02) and GRAL (ANR-10-LABX-49-01) within the Grenoble Partnership for Structural Biology (PSB). We are indebted to Dr JP Kleman with his help on collecting the FRAP data. Part of this work was supported by La Ligue Contre Le Cancer Comité du Rhône (to DS).

## Conflict of interest

The authors declare that the research was conducted in the absence of any commercial or financial relationships that could be construed as a potential conflict of interest.

## Publisher’s note

All claims expressed in this article are solely those of the authors and do not necessarily represent those of their affiliated organizations, or those of the publisher, the editors and the reviewers. Any product that may be evaluated in this article, or claim that may be made by its manufacturer, is not guaranteed or endorsed by the publisher.

## Supplementary material

Supplementary Figure 1
**(A)** Immunofluorescence microscopy images of mitotic cells from unselected naive HCT116 cells and selected HCT116 STLC-resistant cells that were exposed to Eg5 inhibitors, STLC (10μM) K858 (7.5μM), DME (5μM) EgVI (5μM) and GSK-1 (0.5μM) for 18h. Microtubules were detected with an anti-tubulin antibody (green) and chromatin with DAPI (blue). Scale bar in the images corresponds to 10 μm. **(B)** Crystal violet-stained colonies of parental HCT116 cells and STLC-resistant lines (resistant clones R1, R2, R3, R4 and R5) after 14 days exposed to either media or Eg5 inhibitors, STLC (10μM) K858 (7.5μM), DMEI (5μM) EgVI (5μM) and GSK-1 (0.5μM).

Supplementary Figure 2
**(A)** Basal ATPase activity of Eg5(WT) and Eg5(T107N) **(B)** Determination of the optimal microtubule concentration for screening the inhibition of the microtubule-stimulated Eg5 ATPase activity. **(C)** Inhibition of microtubule activated Eg5 ATPase activity of WT-Eg5 (red) and Eg5(T107N) motor domain by STLC.
