# Supplementary figures and images for: Drug resistance dependent on allostery: A P-loop rigor Eg5 mutant exhibits resistance to allosteric inhibition by STLC
Source: Front Oncol. 2022 Oct 12;12:965455. doi: 10.3389/fonc.2022.965455 (PMC9597087; doi:10.3389/fonc.2022.965455)

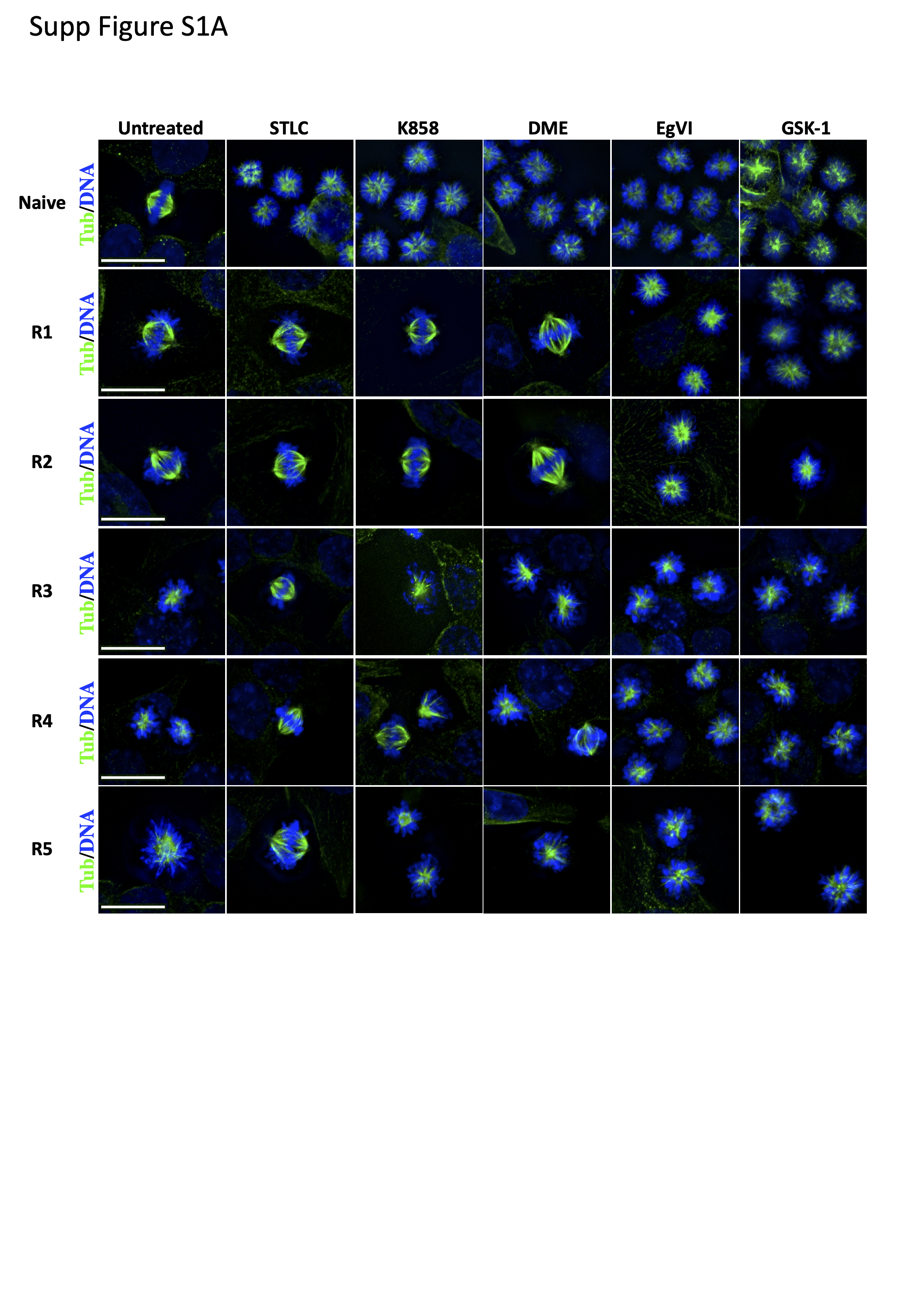

Supplement: Supplementary Figure 1 — (A) Immunofluorescence microscopy images of mitotic cells from unselected naive HCT116 cells and selected HCT116 STLC-resistant cells that were exposed to Eg5 inhibitors, STLC (10μM) K858 (7.5μM), DME (5μM) EgVI (5μM) and GSK-1 (0.5μM) for 18h. Microtubules were detected with an anti-tubulin antibody (green) and chromatin with DAPI (blue). Scale bar in the images corresponds to 10 μm. (B) Crystal violet-stained colonies of parental HCT116 cells and STLC-resistant lines (resistant clones R1, R2, R3, R4 and R5) after 14 days exposed to either media or Eg5 inhibitors, STLC (10μM) K858 (7.5μM), DMEI (5μM) EgVI (5μM) and GSK-1 (0.5μM). [file Image_1.tiff]

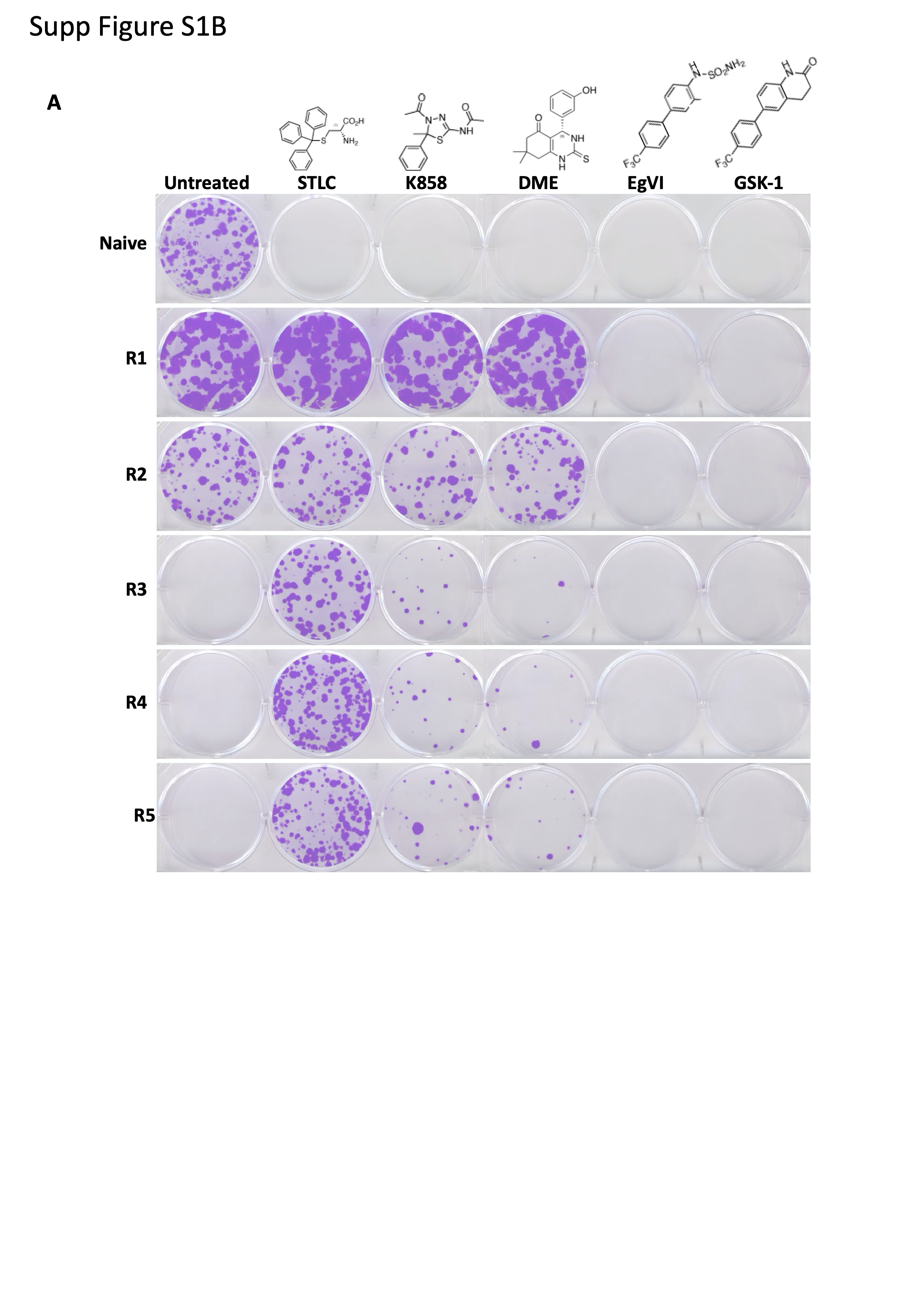

Supplement: Supplementary Figure 2 — (A) Basal ATPase activity of Eg5(WT) and Eg5(T107N) (B) Determination of the optimal microtubule concentration for screening the inhibition of the microtubule-stimulated Eg5 ATPase activity. (C) Inhibition of microtubule activated Eg5 ATPase activity of WT-Eg5 (red) and Eg5(T107N) motor domain by STLC. [file Image_2.tiff]

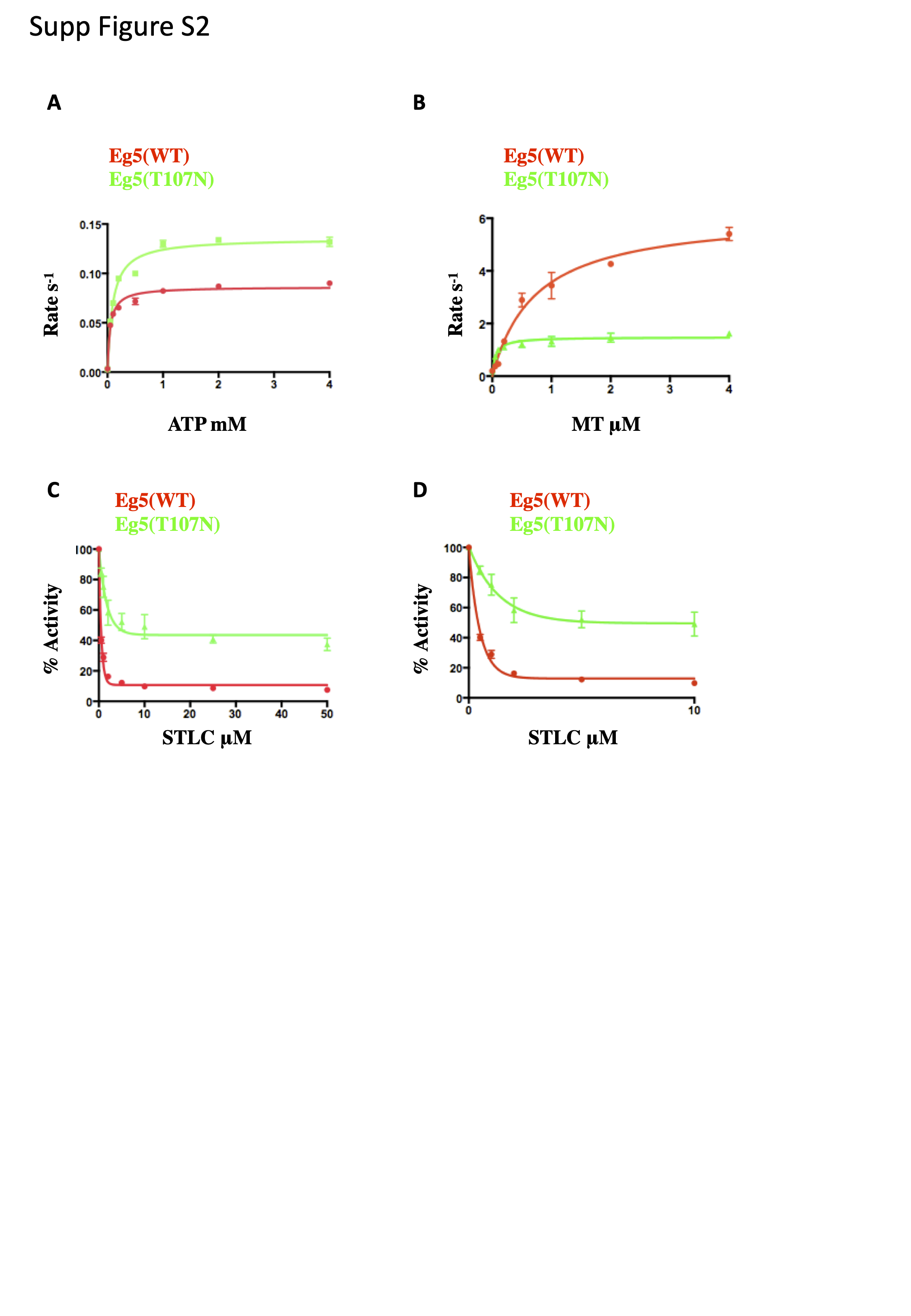

Supplement: Supplementary file 3 [file Image_3.tiff]
